# Baseline Inflammatory Markers as Predictors of Running‐Related Injuries: A One‐Year Prospective 4HAIE Cohort Study

**DOI:** 10.1111/sms.70225

**Published:** 2026-02-12

**Authors:** Lukas Cipryan, Jiri Skypala, Martina Litschmannova, Daniel Jandacka, Tomas Dostal, Dominik Sindler, David Zahradnik, Peter Hofmann

**Affiliations:** ^1^ Department of Human Movement Studies & Human Motion Diagnostic Centre The University of Ostrava Ostrava Czech Republic; ^2^ Department of Applied Mathematics, Faculty of Electrical Engineering and Computer Science VSB – Technical University of Ostrava Ostrava Czech Republic; ^3^ Institute of Human Movement Science, Sport & Health University of Graz Graz Austria

**Keywords:** cytokines, inflammation, injury, running, tumor necrosis factor alpha

## Abstract

Inflammatory processes may contribute to running‐related injury (RRI) susceptibility, yet the predictive value of baseline inflammatory biomarkers remains unclear. This prospective study investigated whether baseline biochemical markers of inflammation predict RRI occurrence in healthy individuals over 1 year, while accounting for training, physiological, and injury history variables. A total of 1315 healthy individuals (recreational runners and inactive controls) were followed for 12 months with prospective injury surveillance. Baseline blood samples were analyzed for inflammatory markers. Multivariable logistic regression examined associations between baseline biomarkers and RRI occurrence, adjusting for age, sex, peak oxygen consumption, weekly running distance, total body fat, and history of musculoskeletal trauma. Significant predictors of RRI included baseline tumor necrosis factor alpha (TNF‐α) (OR per 1 pg/mL: 1.25, 95% CI: 1.08–1.44), history of musculoskeletal trauma (OR: 1.42, 95% CI: 1.06–1.90), weekly running distance, and age. Interleukin‐1 receptor antagonist (IL‐1RA) showed a modest protective association (OR per 10 pg/mL: 0.99, 95% CI: 0.99–1.00). However, the model demonstrated limited discriminatory ability (AUC = 0.66), indicating that baseline inflammatory markers alone are insufficient for individual‐level injury prediction. Baseline TNF‐α and IL‐RA are significantly associated with RRI occurrence, suggesting that inflammatory phenotype contributes to injury susceptibility. However, TNF‐α and IL‐1RA cannot serve as reliable standalone screening tools, and our findings indicate that baseline inflammatory phenotype represents one component of multifactorial injury risk. While baseline TNF‐α and IL‐1RA measurements are unlikely to transform clinical practice in isolation, understanding inflammatory contributions to injury susceptibility may inform more effective injury‐prevention strategies.

## 1 Introduction

Running is a globally practiced form of physical activity associated with substantial health benefits [1, 2]. When appropriately managed, it serves as a cost‐effective strategy for promoting physical fitness [3]. Nevertheless, the incidence of running‐related musculoskeletal injuries (RRIs) remains high. The incidence of RRI among recreational runners observed over an 8‐week training period was 30.1 cases per 1000 h of running exposure [4]. In comparison, an 18‐month cohort study involving 5025 runners demonstrated that the incidence of RRI significantly increased when the distance of a single running session exceeded the runner's typical training distance over the preceding 30 days [5]. Among the most frequently occurring new RRIs are Achilles tendinopathy, medial tibial stress syndrome, patellofemoral pain syndrome, plantar fasciitis, and ankle sprains [6]. RRIs may be associated with localized inflammation, which plays a critical role in the body's response to tissue damage and the subsequent healing processes [7].

Inflammation represents a fundamental aspect of the innate immune response, playing a critical role in promoting organismal survival during pathogenic invasion or tissue injury, while also maintaining tissue homeostasis under various adverse environmental conditions. Typically, this process is acute and localized. However, when inflammatory stimuli persist or resolution fails, the response may shift toward a chronic state [8]. Chronic inflammation can be systemic and often manifests as low‐grade, subclinical activation of immune pathways. This low‐grade chronic systemic inflammation has been increasingly recognized as a pivotal risk factor in the etiology and progression of numerous chronic diseases, including obesity, cancer, diabetes mellitus, chronic kidney disease, non‐alcoholic fatty liver disease, cardiovascular disorders, and autoimmune and neurodegenerative conditions, thereby significantly impacting global morbidity and mortality [8, 9, 10]. Emerging evidence suggests it may also predispose individuals to RRIs [11, 12].

Cytokines, typically polypeptides or glycoproteins, mediate autocrine and paracrine signaling with both pro‐inflammatory and anti‐inflammatory effects. They regulate a broad spectrum of biological processes in target cells, including functional activity, differentiation, proliferation, apoptosis, and survival [13]. In response to physical exercise, cytokine expression is upregulated, contributing to the initiation, modulation, or resolution of inflammation relevant to musculoskeletal health [7]. Exercise‐induced inflammation constitutes a fundamental physiological mechanism that supports recovery, tissue regeneration, and adaptation to exercise stress. Pro‐inflammatory cytokines are essential for the clearance of cellular debris and the initiation of the healing process, whereas anti‐inflammatory cytokines facilitate tissue repair through interactions with various cell types, thereby promoting muscle adaptation and hypertrophy [14].

Therefore, we hypothesize that baseline inflammatory phenotype, as reflected by cytokine levels, may identify individuals at higher risk for RRIs, especially those caused by overuse and repetitive strain. We specifically investigated whether single‐point baseline measurements of inflammatory markers could serve as predictive indicators of injury susceptibility rather than markers of active pathological processes at the time of injury occurrence. This prospective study aimed to elucidate the associations between biochemical markers of inflammation and the onset of RRIs in a large cohort of healthy individuals, while accounting for the multifactorial etiology of RRIs. These injuries are influenced by a complex interplay of training characteristics, health and lifestyle factors, as well as morphological and biomechanical variables [15, 16], however, may be triggered by previous low‐grade inflammation. To mitigate potential confounding, a multivariate logistic regression model was employed, incorporating key covariates such as age, sex, and weekly running volume. Furthermore, given that previous injury is widely recognized as the strongest predictor of future injury, the analyses also examined and statistically controlled for the influence of prior musculoskeletal injuries.

## 2 Materials and Methods

### 2.1 Study Design

The presented study is part of the multidisciplinary research project “Healthy Aging in Industrial Environment – Programme 4” (4HAIE), which investigates the long‐term influence of ambient air pollution on health of runners and inactive individuals [17, 18, 19]. This one‐year prospective investigation focuses on reported running‐related injuries onset (dependent variable) and baseline biochemical markers of inflammation measured at study entry (independent variables) as potential predictive variables. Blood samples were collected at baseline (study entry) to establish participants´ inflammatory phenotypes prior to the one‐year injury surveillance period. This design allowed investigation of whether baseline biomarker profiles could predict future injury occurrence. We considered also other covariates: age, sex, $\dot{V}$O_2peak_, average running distance per week, total body fat (%), and retrospective musculoskeletal trauma (MST). The selection of covariates reflects the multifactorial nature of running‐related injuries, as evidenced by recent systematic reviews and prospective cohort studies [15, 16, 20]. Incorporating these variables into the analysis enables control for key demographic, physiological, and training‐related risk factors that have been shown to contribute to the occurrence of RRIs.

### 2.2 Participants

Participants were Caucasian adults (*N* = 1315; age 18–65 years). Participants, including both runners and physically inactive individuals, were recruited from two distinct geographic regions characterized by differing levels of air pollution, in accordance with the primary objectives of the original research project 4HAIE. No statistically significant differences in the incidence of RRIs were observed between the two regions.

Inclusion criteria—runners:

Running as a main exercise activity > 150 min of moderate or > 75 min of strenuous physical activity per week (or an equivalent combination of moderate and strenuous physical activity) [21], > 10 km running per week for at least 6 weeks prior to the tests, intending to continue running for next 12 months, permanent (> 5 years) whole‐year residency in the determined areas, not planning to move away from the determined areas during the next 12 months, with internet access, using a smart phone (with iOS or Android 5.0 or higher).

Inclusion criteria—inactive individuals:

< 150 min of moderate or < 75 min of strenuous exercise per week, capable of running, but running irregularly and/or less than 6 weeks prior to the tests, no contraindications to exercise, permanent (> 5 years) whole‐year residency in the determined areas, not planning to move away from the determined areas during the next 12 months, with internet access, using a smart phone (with iOS or Android 5.0 or higher).

Exclusion criteria—runners and inactive individuals:

Acute (within 6 weeks) health condition (pain, injury, surgery) preventing from physical activity, any other acute disease, pregnancy, radiological examination within the last 7 days, artificial pacemaker, radioactive, surgical or any other device/implant, insulin pump, smoking.

All procedures were conducted in compliance with relevant institutional guidelines and regulatory requirements. The 4HAIE study adheres to the principles outlined in the Declaration of Helsinki. The study protocol received approval from the Ethics Committee of the local university (approval number: 3/2018). Prior to enrolment, each participant is provided with a comprehensive information sheet and is required to give written informed consent. The study involves minimal to no risk to participants.

### 2.3 Anthropometry

Anthropometric measurements included basic anthropometric parameters (body height and body mass) and body composition. All measurements were taken in the morning. The participants were measured in sports clothing (shorts and T‐shirt) and barefoot. The standard conditions for the bioelectrical impedance analysis (BIA) method measurements were provided by the specific schedule of the study protocol. All participants were housed in supervised accommodation at the research centre about 15 h prior to the BIA measurement which supported the standard requirements before the measurement. Body height was measured first using the InBody 370 stadiometer (Biospace, South Korea), followed by body mass and hydration status (total body water, intracellular and extracellular water), which were measured using the InBody 770 bioimpedance multifrequency scale device (Biospace, South Korea).

### 2.4 Cardiorespiratory Fitness (CRF) Assessment

Participants performed a graded exercise test (GXT) on a motorized treadmill (Rodby RL 2500E) to determine peak aerobic power ($\dot{V}$O_2peak_). Prior to the GXT, participants completed 3 min of walking at 5.0 km/h to familiarize themselves with the treadmill. The GXT protocol then started at 6.0 km/h, with speed subsequently increasing by 1.0 km/h every minute (inclination remaining at 1%) until volitional exhaustion. Expired air was continuously monitored to analyze O_2_ and CO_2_ concentrations during the GXT with a breath‐by‐breath system (Blue Cherry, Geratherm Medical AG, Germany). The highest average O_2_ consumption measured over a 30 s period was used to determine $\dot{V}$O_2peak_ [22]. Perceived effort was obtained using the 20‐point Borg scale. All sessions were conducted in the afternoon, at least 3 h after the participants' last meal and in a thermally controlled laboratory (21°C, 40% relative humidity). Each participant was advised not to participate in any vigorous activity 24 h prior to the test. Participants who did not pass the Physical Activity Readiness Questionnaire (PAR‐Q) were not allowed to perform the GXT unless explicit permission given by a medical doctor was provided. Blood pressure (BP) was also checked before the GXT. In case of BP values ≥ 150/90 mmHg, participants without a medical permission were not allowed to perform the GXT but continued in the study protocol. Detailed results for exercise performance have been presented recently [23].

### 2.5 Blood Analysis

Fasting blood samples were collected from the antecubital vein at the start of the one‐year observation period. Whole blood samples with EDTA as an anticoagulant were used immediately for blood count and glycated hemoglobin (HbA1c) determination. Whole blood samples for fibrinogen analysis were collected separately in tubes with sodium citrate and centrifuged at 2500 g for 15 min to separate plasma. Serum collection tubes were allowed to clot for 30 min and subsequently centrifuged at 2500 g for 10 min to separate the serum. Blood serum was divided into five 200–500‐μl aliquots, which were frozen at −80°C until analysis. The S‐Monovette (Sarstedt, Nümbrecht, Germany) and Vacuette systems (BD, Mississauga, Canada) were used for blood sample collection.

HbA1c was measured using a Tosoh G11 (Tosoh, Tokyo, Japan). Glucose, triglyceride (TG), total cholesterol, high‐ and low‐density lipoprotein cholesterol (HDL‐C and LDL‐C, respectively), and C‐reactive protein (CRP) concentrations were measured using a Cobas 8000 device (Roche, Basel, Switzerland).

Cytokines and growth factor determinations were performed in a series of runs with different batches of kits so that the expiration of the kits was not exceeded. This analysis was performed by the ALBIA (addressable laser bead assay) technique and the tests performed were measured and evaluated on a Luminex 200. The following R&D Systems kits supplied by Bio‐Techne R&D Systems were used: LXSAHM‐01 Adiponectin, LXSAHM‐01 brain‐derived neurotrophic factor (BDNF) and LXSAHM‐06 interleukin‐1β (IL‐1β), interleukin‐1 receptor antagonist (IL‐1RA), interleukin‐6 (IL‐6), interleukin‐10 (IL‐10), tumor necrosis factor alpha (TNFα) and leptin. For Discovery Luminex kits, the manufacturer reports general intra‐assay coefficients of variation (CVs) < 20% and inter‐assay CVs < 30%; per‐analyte CVs are not provided. Precision depends on assay execution and sample handling. In practice, CVs are typically lower. For CRP and fibrinogen, intra‐ and inter‐assay CVs were < 5%.

As part of the preprocessing, null values of numerical variables (results of blood analysis) that corresponded to measurements below the detection limit were imputed as one‐half of the detection limit.

### 2.6 Retrospective Musculoskeletal Trauma (MST)

Retrospective data on musculoskeletal trauma were collected during the baseline assessment at the onset of the one‐year study. Information was obtained using three complementary methods. First, participants completed a demographic questionnaire that included items pertaining to their running history. Second, they filled out the standardized Running Injuries Survey Flow [24], which assessed prior running‐related injuries and current running status. Third, a structured interview was conducted by a physiotherapist as part of the baseline functional assessment, focusing on participants' medical history and previous musculoskeletal issues. All reported instances of retrospective musculoskeletal trauma, regardless of the source, were included in the final data analysis. The basic characteristics are presented in the (Tables S3 and S4).

### 2.7 Running‐Related Injuries (RRIs)

Following the baseline assessment, participants underwent a one‐year observation period that included monitoring of RRIs. A running‐related injury was defined as musculoskeletal pain located in the lower extremities or lower back that required medical attention or led to a reduction or cessation of running activity (distance, speed, duration, or training load) for at least 7 days or across three consecutive scheduled running sessions [25]. Based on this definition, participants were instructed to self‐report RRIs throughout the one‐year follow‐up period. These injuries were documented using a standardized questionnaire [26] delivered through a mobile application developed specifically for the 4HAIE cohort study. Throughout the entire 12‐month observation, each participant was instructed to continuously wear a FitBit Charge 3 device for the purpose of monitoring habitual physical activity levels. Injury questionnaires could be completed through three modes: (1) a self‐initiated reporting when experiencing pain or suspected injury, (2) a scheduled weekly injury questionnaire automatically distributed every Sunday between 4:00 and 8:00 PM, and (3) a triggered questionnaire when Fitbit‐derived activity levels fell below the individual's usual weekly activity, prompting participants to confirm whether the decrease was injury‐related [19]. Further details regarding the one‐year monitoring framework of the cohort are described in the published 4HAIE protocol [19].

### 2.8 Statistical Analysis

All analyses were performed using all observations with complete data for the variables included in each model using the R software (version 4.0.2, www.r‐project.org), and the significance level was set to 0.05.

These analyses were conducted to address the exploratory aim of describing differences between the injured and uninjured groups, complementing the study's primary aim of identifying predictors of injury onset through multivariable modeling.

In the first step, the basic characteristics of the cohort and the descriptive characteristics of the variables used were calculated. Categorical variables are presented as absolute frequencies and relative frequencies in percentages (in brackets). For a univariate description of numerical variables, the minimum (Min), maximum (Max), median (M), and interquartile range (IQR) were reported, as several distributions exhibited meaningful deviations from normality, even in a large sample according to the Shapiro–Wilk test, visual assessment, and the identification of outliers.

Descriptive and inferential analyses were performed to compare participants who sustained an RRI with those who did not, using descriptive statistics and Mann–Whitney U tests for continuous variables (applied due to the non‐normal distribution of several variables). Because descriptive group comparisons were used for cohort characterization rather than inferential decision‐making, *p*‐values were not adjusted for multiple comparisons; the primary conclusions are based on the multivariable model. For between‐group comparisons, effect sizes were quantified using effect size r, which is appropriate for the Mann–Whitney U test and reflects the magnitude of group differences.

Second, to analyze the influence of selected factors on the occurrence of RRI, logistic regression was employed. Logistic regression was used to model the association between predictors and the binary outcome (RRI onset). Biomarker values were analyzed on their original scale to preserve clinical interpretability; although log‐transformation can reduce skewness, it was not required for logistic regression and is noted as a methodological consideration.

Based on prior knowledge of associations between the examined factors and injury risk [6, 16, 27], as well as the results of descriptive statistics and inferential testing, the following variables were selected for inclusion in the initial regression model: Age (years), Sex, Prospective running distance from Fitbit Charge (km/week), Retrospectively reported musculoskeletal trauma (MST), *V̇*O₂peak (ml/kg/min), Total body fat (%), IL‐1β (pg/ml), IL‐1RA (pg/ml), hs‐IL‐6 (pg/ml), IL‐10 (pg/ml), TNF‐α (pg/ml), CRP (mg/l), Fibrinogen (g/l), Adiponectin (ng/ml), Leptin (pg/ml), Adiponectin/Leptin ratio (−), and BDNF (pg/ml).

Due to a strong negative correlation between *V̇*O₂peak and total body fat (Pearson's ρ (95% CI): −0.813 (−0.832; −0.793)), variance inflation factors (VIFs) were calculated to assess multicollinearity. A VIF of 1 indicates no correlation, whereas values above 5 are commonly interpreted as a warning sign of potentially problematic multicollinearity. In our case, both *V̇*O₂peak (VIF = 5.93) and total body fat (VIF = 5.17) exceeded this threshold. Together with the high absolute value of the correlation coefficient and the visual inspection of the correlation scatterplot, these findings indicated that including both variables would likely introduce redundancy and reduce model stability. Although total body fat is biologically relevant, *V̇*O₂peak was retained as the physiologically more integrative measure. Therefore, total body fat was excluded from the final set of predictors. No indications of multicollinearity were observed among the remaining variables.

The initial full model was specified a priori based on established risk factors reported in previous literature, and a reduced model was subsequently presented for clarity, including only predictors that remained significant in the full model; both models yielded consistent results, reducing concerns about data‐driven selection.

## 3 Results

### 3.1 Study Population

The results of the multivariate logistic regression analysis included data from 1159 participants. An additional 156 individuals were excluded due to missing data or failure to complete the one‐year follow‐up. The most commonly missing data were *V̇*O_2peak_ values resulting from the inability to complete GXT because of elevated blood pressure. Descriptive characteristics of this selected cohort are summarized in Tables 1 and 2. Characteristics of the full cohort (*N* = 1315) are provided in the (Table S1).

**TABLE 1:** Basic characteristics of the cohort (*N* = 1159; male/female: 542 (46.8%)/617 (53.2%)).

	(min; max)	M (Q1; Q3)
Age (years)	(18.0; 65.0)	38.0 (27.0; 46.0)
Height (cm)	(148.5; 201.6)	174.5 (167.8; 181.3)
Body mass (kg)	(40.5; 123.6)	73.6 (63.7; 84.0)
BMI (kg/m^2^)	(16.0; 43.0)	23.9 (21.7; 26.4)
Total body fat (%)	(3.5; 50.2)	20.5 (15.2; 27.6)
*V̇*O_2peak_ (ml/kg/min)	(14.6; 70.9)	41.9 (34.8; 49.5)
*V̇*O_2peak_ (l/min)	(1.14; 5.67)	3.00 (2.40; 3.81)
RER (−)	(0.62; 2.23)	1.12 (1.07; 1.16)
Running distance (km/week)	(0.1; 95.2)	5.5 (0.7; 13.7)
Systolic BP (mmHg)	(84.0; 180.0)	126.0 (116.0; 134.0)
Diastolic BP (mmHg)	(45.3; 111.7)	78.0 (72.0; 84.7)
IL‐1β (pg/ml)	(0.00; 63.17)	0.39 (0.19; 0.51)
IL‐1RA (pg/ml)	(0.2; 6235.1)	471.6 (346.8; 623.2)
IL‐6 (pg/ml)	(0.00; 145.77)	0.17 (0.07; 0.54)
IL‐10 (pg/ml)	(0.00; 64.47)	0.12 (0.06; 0.40)
TNF‐α (pg/ml)	(0.02; 10.89)	0.90 (0.38; 1.53)
CRP (mg/l)	(0.50; 55.10)	0.50 (0.50; 1.70)
Fibrinogen (g/l)	(0.45; 5.00)	2.60 (2.29; 2.95)
Adiponectin (ng/ml)	(2; 106 189)	6006 (3814; 9017)
Leptin (pg/ml)	(1; 92 026)	6011 (2391; 12 052)
Adiponectin/Leptin (−)	(0.00; 8570.14)	1.01 (0.42; 2.60)
BDNF (pg/ml)	(1; 142 792)	24 151 (18 829; 29 830)

Abbreviations: BDN, brain derived neurotrophic factor; BMI, body mass index; BP, blood pressure; CRP, C‐reactive protein; IL‐10, interleukin 10; IL‐1RA, interleukin 1 receptor antagonist; IL‐1β, interleukin 1β; IL‐6, interleukin 6; M, Median; Q1/Q3, quartile 1 and 3; RER, respiratory exchange ratio; TNF‐α, tumor necrosis factor α; V̇O_2peak_, peak oxygen consumption.

**TABLE 2:** Basic characteristics of participants with/without RRIs.

	RRIs (*N* = 314; male/female: 185 (58.9%)/129 (41.1%))		No RRIs (*N* = 845; male/female: 432 (51.1%)/413 (48.9%))		Mann–Whitney test	
	(min; max)	M (Q1; Q3)	(min; max)	M (Q1; Q3)	*p*	ES (95% CI)
Age (years)	(18.0; 64.0)	40.0 (30.0; 46.0)	(18.0; 65.0)	37.0 (25.0; 46.0)	**0.008**	0.078 (0.020; 0.130)
Height (cm)	(153.6; 198.5)	175.5 (168.6; 182.4)	(148.5; 201.6)	174.0 (167.5; 181.1)	**0.025**	0.066 (0.010; 0.120)
Body mass (kg)	(44.6; 121.1)	73.9 (65.1; 82.7)	(40.5; 123.6)	73.5 (63.1; 84.5)	0.573	0.017 (0.001; 0.070)
BMI (kg/m^2^)	(17.36; 39.81)	23.80 (21.64; 25.62)	(15.97; 42.95)	23.97 (21.71; 26.75)	0.544	0.018 (0.001; 0.080)
Total body fat (%)	(3.5; 49.7)	20.1 (14.3; 25.4)	(3.9; 50.2)	20.9 (15.5; 28.2)	**0.002**	0.089 (0.030; 0.140)
*V̇*O_2peak_ (ml/kg/min)	(17.7; 70.9)	43.2 (37.0; 50.5)	(14.6; 70.6)	40.4 (33.6; 48.5)	**< 0.001**	0.112 (0.050; 0.170)
*V̇*O_2peak_ (l/min)	(1,28; 5.67)	3.35 (2.53; 3.94)	(1.14; 5.61)	2.95 (2.33; 3.73)	**< 0.001**	0.119 (0.060; 0.170)
RER (−)	(0.92; 1.77)	1.11 (1.06; 1.15)	(0.62; 2.23)	1.12 (1.07; 1.17)	**0.006**	0.080 (0.020; 0.140)
Running distance (km/week)	(0.12; 95.18)	9.88 (3.53; 19.13)	(0.10; 77.36)	3.53 (0.58; 11.55)	**< 0.001**	0.236 (0.180; 0.290)
Systolic BP (mmHg)	(95.7; 180.0)	127.0 (118.3; 136.0)	(84.0; 173.0)	125.7 (116.0; 134.0)	**0.033**	0.063 (0.007; 0.110)
Diastolic BP (mmHg)	(57.3; 104.7)	79.0 (72.1; 84.6)	(45.3; 111.7)	78.0 (71.7; 84.7)	0.232	0.035 (0.001; 0.100)
IL‐1β (pg/ml)	(0.00; 10.22)	0.45 (0.25; 0.60)	(0.00; 63.17)	0.39 (0.19; 0.51)	**0.011**	0.075 (0.020; 0.140)
IL‐1RA (pg/ml)	(0.2; 2481.2)	445.5 (318.7; 583.0)	(0.2; 6235.1)	480.2 (358.7; 640.8)	**0.003**	0.088 (0.030; 0.150)
IL‐6 (pg/ml)	(0.00; 145.77)	0.23 (0.09; 0.70)	(0.00; 25.54)	0.15 (0.07; 0.51)	0.065	0.054 (0.004; 0.110)
IL‐10 (pg/ml)	(0.00; 5.52)	0.15 (0.05; 0.41)	(0.00; 64.47)	0.12 (0.06; 0.40)	0.655	0.013 (0.001; 0.070)
TNF‐α (pg/ml)	(0.02; 8.48)	0.96 (0.38; 1.70)	(0.04; 10.89)	0.90 (0.38; 1.43)	**0.030**	0.064 (0.007; 0.120)
CRP (mg/l)	(0.50; 21.60)	0.50 (0.50; 1.60)	(0.50; 55.10)	0.50 (0.50; 1.80)	0.132	0.044 (0.003; 0.100)
Fibrinogen (g/l)	(0.45; 4.14)	2.58 (2.29; 2.92)	(1.52; 5.00)	2.61 (2.30; 2.97)	0.251	0.034 (0.002; 0.090)
Adiponectin (ng/ml)	(7; 20 408)	6376 (4255; 9833)	(2; 106 189)	5780 (3729; 8639)	**0.029**	0.068 (0.007; 0.130)
Leptin (pg/ml)	(1.4; 75 127)	4783 (1851; 10 035)	(4; 92 026)	6574 (2604; 13 184)	**0.014**	0.072 (0.010; 0.130)
Adiponectin/Leptin (−)	(0.00; 8570.14)	1.38 (0.61; 3.17)	(0.00; 1524.62)	0.89 (0.37; 2.39)	**< 0.001**	0.136 (0.080; 0.190)
BDNF (pg/ml)	(1; 117 150)	24 810 (19 231; 30 379)	(247; 142 792)	23 790 (18 786; 30 378)	0.150	0.042 (0.002; 0.100)

Abbreviations: BDNF, brain derived neurotrophic factor; BMI, body mass index; BP, blood pressure; CRP, C‐reactive protein; ES (95% CI), Effect Size (95% Confidence Interval); IL‐10, interleukin 10; IL‐1RA, interleukin 1 receptor antagonist; IL‐1β, interleukin 1β; IL‐6, interleukin 6; M, Median; Q1/Q3, quartile 1 and 3; RER, respiratory exchange ratio; TNF‐α, tumor necrosis factor α; V̇O_2_peak, peak oxygen consumption.

### 3.2 Descriptive Statistics and Between‐Group Comparisons

Basic characteristics of the cohort (*N* = 1159) are presented in Table 1. Table 2 compares the same variables between participants who did (*N* = 314; 27.1%) and did not (*N* = 845; 72.9%) report a running‐related injury during the observation period. Differences were evaluated using the Mann–Whitney U test. Several variables, including age, *V̇*O₂peak, running distance, total body fat, IL‐1β, IL‐1RA, TNF‐α, adiponectin, leptin, and the adiponectin/leptin ratio, differed significantly between the two groups, although effect sizes were generally small.

### 3.3 Biochemical Markers of Inflammation and Other Risk Markers

A full multivariate logistic regression (Model 0; see Table S2) was first performed using all candidate predictors. The selection of these predictors is described in detail in the Statistical Analysis section. Subsequently, a reduced model (Model 1; see Table 3) was fitted including only those variables that remained significant. The two models did not differ significantly (*p* = 0.168). Significant predictors of RRI in Model 1 were retrospective MST, age, average running distance per week, TNF‐α, and IL‐1RA, the latter demonstrating a protective association.

**TABLE 3:** Multivariate logistic regression for the significant predictors (Model 1).

Predictors	Coef	SE (Coef)	*p*	OR (95% CI)	
Intercept	−1.690	0.268	< 0.001	—	
Retrospective MST (Yes/No)	0.434	0.139	0.001	1.54 (1.18, 2.03)	
Age (years)	0.010	0.006	0.078	1.01 (1.00, 1.02)	
Running distance (km/week)	0.026	0.005	< 0.001	1.03 (1.02, 1.04)	
IL‐1RA (pg/ml)	−0.001	0.000	0.004	1.00 (1.00, 1.00)	0.99 (0.99, 1.00)*
TNF‐α (pg/ml)	0.220	0.073	0.002	1.25 (1.08, 1.44)	1.02 (1.01, 1,04)**

*Note: n* = 1159, *R* ^2^ = 0.060, AIC = 1296.6.

Abbreviations: CI, confidence interval; IL‐1RA, interleukin receptor antagonist; OR, odds ratio; Retrospective MST, Retrospective musculoskeletal trauma; TNF‐α, tumor necrosis factor α.

* OR for 10 pg/ml change.

** OR for 0.1 pg/ml change (clinically more relevant interpretations).

Although the odds ratios for age, running distance, and IL‐1RA were modest, baseline TNF‐α showed predictive value, i.e., each 1 pg/mL increase was associated with 25% higher odds of subsequent RRI occurrence (95% CI: 8%–44%) during the follow‐up period, after adjusting for other predictors. These associations reflect the predictive capacity of baseline inflammatory markers rather than inflammatory status at the time of injury. Retrospective MST was significantly associated with RRI, with an odds ratio of 1.42 (95% CI: 1.06–1.90). The overall discriminatory ability of Model 1 was poor, as indicated by an area under the ROC curve (AUC) of 0.66 and Nagelkerke R [2], which was 0.055.

Further modeling of the separate effect of each significant variable (age, running distance, IL‐1RA, Retrospective MST, TNF‐α) on RRIs (i.e., without adjusting for other covariates) yielded similar results (data not shown). Importantly, the odds ratio for TNF‐α remained significant (*p* = 0.003; OR (95% CI): 1.24 (1.07, 1.44)).

To explore the robustness of the findings, we conducted a set of sensitivity analyses using alternative definitions of the outcome variable based on the nature and timing of injury reports. RRIs were categorized as: (1) self‐reported only (*N* = 215), (2) medically confirmed by a physician (*N* = 171), and (3) reported within 30 days after baseline blood collection (*N* = 89). While age, running distance, and IL‐1RA showed statistical significance in multiple definitions, a consistently substantial association was observed only for TNF‐α:

1. *p* = 0.022, OR (95% CI): 1.20 (1.02, 1.40).
2. *p* = 0.003, OR (95% CI): 1.27 (1.08, 1.50).
3. *p* = 0.094, OR (95% CI): 1.19 (0.95, 1.46).

We additionally performed a subgroup analysis including only runners to verify whether the identified predictors remained consistent after excluding physically inactive individuals. Retrospective MST (*p* = 0.001, OR (95% CI): 1.72 (1.23–2.40)) and TNF‐α (*p* = 0.001, OR (95% CI): 1.38 (1.14–1.68)) retained statistical significance.

These results are consistent with the primary analysis and may serve as a sensitivity check supporting the robustness of the association between TNF‐α and RRIs. Detailed group comparisons of retrospectively reported MST and RRI for both the full and the reduced cohort are presented in Supplementary Tables S3 and S4.

## 4 Discussion

This prospective observational study examined the associations RRIs reported over a one‐year period and biochemical markers of inflammation in a cohort comprising both healthy runners and inactive individuals at the start of the observation period. In addition to inflammatory biomarkers, relevant covariates including age, sex, *V̇*O₂peak, total body fat, and average running distance per week were included in the multivariate regression analyses. Our principal finding was that baseline TNF‐α emerged as a significant predictor of RRI occurrence, though with important limitations regarding its clinical utility.

We identified baseline TNF‐α as a significant predictor of RRI occurrence during one‐year follow‐up. Specifically, each 1 pg/mL increase in baseline TNF‐α concentration, representing a substantial change within the typical physiological range of approximately 5.5 pg/mL (95% CI 3.8–8.0 pg/mL) in healthy individuals [28], was associated with a 25% increase in the odds of sustaining an RRI occurrence (95% CI: 8%–44%), after adjusting for other covariates. For a more cautious clinical interpretation, recalculating the effect per 0.1 pg/mL increase indicates that each 0.1 pg/mL rise in TNF‐α is associated with a 2% (95% CI: 1%–4%) increase in the odds of RRI. However, further assessment of the model's predictive performance indicated that its overall discriminatory ability was limited. The model thus lacks sufficient statistical power to support the use of TNF‐α as a reliable standalone predictor of RRI incidence in practical applications. To understand why TNF‐α shows this association despite limited predictive power, we must consider how baseline inflammatory status relates to musculoskeletal injury susceptibility in the context of running‐induced mechanical stress.

The observed association between baseline TNF‐α and RRI risk may reflect individual differences in inflammatory phenotype that become clinically relevant under repetitive mechanical load. Unlike acute exercise responses, which temporarily elevate TNF‐α as part of normal adaptation [29], our findings suggest that constitutively higher baseline levels may indicate a predisposition to maladaptive inflammatory responses. This interpretation aligns with evidence that TNF‐α plays a dual role in musculoskeletal adaptation: transient elevation supports tissue remodeling, whereas sustained elevation impairs muscle regeneration and promotes tissue degradation [11, 12, 30, 31]. In our cohort, runners with higher baseline TNF‐α may therefore be less capable of mounting effective repair responses to the cumulative microtrauma inherent in distance running, ultimately manifesting as increased injury occurrence over the one‐year follow‐up period. This mechanistic interpretation must be reconciled with the limited predictive accuracy (AUC = 0.66) observed in our model, which highlights the multifactorial nature of RRI etiology.

The modest discriminatory ability of our model, despite the statistical significance of TNF‐α, underscores that baseline inflammatory status represents only one component of a complex injury causation pathway. RRIs arise from the interaction of multiple domains: biomechanical factors (running mechanics, footwear), training variables (volume, intensity, progression), anatomical characteristics (alignment, prior injury), and physiological responses (tissue adaptation capacity, recovery efficiency). Our finding that TNF‐α contributes meaningful but insufficient predictive information suggests that inflammatory phenotype modulates—rather than determines—injury risk. This is consistent with the broader understanding that overuse injuries result from a mismatch between cumulative mechanical load and tissue tolerance, where baseline inflammation may shift the threshold at which this mismatch becomes pathological but does not act as a singular causative factor. Beyond TNF‐α, we also examined whether other inflammatory markers demonstrated associations with RRI occurrence in our cohort.

We analyzed additional cytokines and biochemical markers hypothesized to associate with RRIs, including pro‐inflammatory cytokines (IL‐1β) and anti‐inflammatory mediators (IL‐6, IL‐10, IL‐1RA), all known to be modulated by exercise [7]. With the exception of IL‐1RA, none demonstrated meaningful associations with RRI occurrence in our cohort. The negative findings for most inflammatory markers warrant careful interpretation: they may reflect either (1) true absence of association at baseline measurement, (2) insufficient sensitivity of single‐timepoint assessment to capture relevant inflammatory dynamics, or (3) predominance of non‐inflammatory mechanisms in RRI pathogenesis within our population. Notably, IL‐1RA showed a statistically significant negative association with RRI onset, though the effect size was modest. Given that physiological IL‐1RA levels in healthy adults range from approximately 270–700 pg/ml [32, 33, 34], we calculated that each 10 pg/mL increase corresponds to OR 0.99 (95% CI: 0.99; 1.00), representing an approximately 1% decrease in injury odds. While this suggests potential protective effects of higher anti‐inflammatory capacity, the clinical significance remains uncertain given the small magnitude and the limited overall model performance. Importantly, the contrasting directions of association—TNF‐α as a risk factor and IL‐1RA as potentially protective—align with their opposing biological roles and support the biological plausibility of our findings, even though neither marker achieves clinically useful predictive accuracy in isolation. The interpretation of these findings must be considered in light of both the strengths and limitations of our study design.

## 5 Strength and Limitations

Several methodological considerations affect the interpretation and generalizability of our results. A major strength of this study lies in its large cohort, stratified by age decades, which enables age‐specific analyses. Furthermore, the comprehensive multivariate assessment incorporating prospectively reported RRIs, cardiorespiratory fitness, body composition, sex, age, and selected biochemical markers of inflammation and chronic disease enhances the robustness of the findings by allowing for adjustment for multiple potential confounders. The analysis of RRIs across different reporting modalities, i.e., self‐reported, physician‐confirmed, and those reported within 30 days following baseline blood collection, consistently demonstrated a significant association between RRIs and TNF‐α, supporting the validity of the findings and serving as a sensitivity analysis. Additionally, another sub‐analysis was conducted exclusively on the subgroup of runners, and across all these sensitivity analyses, the associative relationship between RRIs and TNF‐α was confirmed. An additional strength of the study lies in its consideration of prior musculoskeletal injuries, including both mild and severe cases affecting the back and lower limbs.

However, several important limitations warrant acknowledgment. Most critically, although our study employed a prospective cohort design with a one‐year follow‐up for injury occurrence, biochemical markers were assessed only at baseline. The single‐timepoint design, while appropriate for investigating whether baseline inflammatory phenotyping could serve as a practical screening tool, precludes assessment of temporal dynamics between inflammation and injury development and limits causal inference. Moreover, single‐timepoint cytokine measurements may be influenced by circadian variation, recent training, subclinical infections, menstrual cycle phase [35], and dietary patterns [36, 37]—factors we did not systematically control, potentially attenuating or biasing observed associations. To address these limitations, a longitudinal follow‐up study with serial biomarker measurements has been initiated, though results are not yet available. Secondly, the absence of a significant association between RRIs and other pro‐inflammatory markers may be questioned, considering the multifaceted nature of inflammatory pathways. In particular, the mechanistic involvement of TNF‐α in this context requires further elucidation. Thirdly, additional training‐related characteristics, such as exercise intensity and weekly training frequency, were not accounted for, although these factors may significantly influence inflammatory responses and injury risk. Identifying the optimal range of physical activity is essential not only for minimizing the risk of overuse injuries but also for maximizing the overall health benefits associated with endurance exercise. Fourthly, the multivariable model included 1159 out of 1315 participants; 156 participants were excluded, primarily due to missing *V̇*O_2peak_ values. In most cases, *V̇*O_2peak_ was unavailable because elevated blood pressure at baseline precluded the administration of the GXT. This exclusion criterion introduces the possibility of selection bias, as participants with higher blood pressure may differ systematically in both exposure variables and outcomes compared to those who completed GXT. Consequently, the analytic sample may underrepresent individuals with certain cardiovascular risk profiles, potentially limiting the generalizability of our findings. Fifthly, although total body fat was excluded due to collinearity with *V̇*O_₂peak_, *V̇*O_₂peak_ itself was non‐significant, making it unlikely that substituting total body fat would have changed the results; however, adiposity remains biologically relevant and warrants further investigation using alternative modeling approaches. Lastly, the cohort exhibits an educational bias, with a disproportionate representation of individuals possessing higher levels of educational attainment. Despite these limitations, our findings offer several implications for clinical practice and future research.

## 6 Conclusion

This study demonstrates that baseline TNF‐α is significantly associated with RRI occurrence over one‐year follow‐up, with each 1 pg/mL increase conferring a 25% increase in injury odds after adjustment for relevant covariates. However, the limited discriminatory ability of our predictive model (AUC = 0.66) indicates that TNF‐α cannot serve as a reliable standalone screening tool in its current application. Rather, our findings suggest that baseline inflammatory phenotype represents one component of multifactorial injury risk, potentially useful for risk stratification when integrated with biomechanical, training‐related, and anatomical variables. The observation that IL‐1RA showed a modest protective association, opposite in direction to TNF‐α, supports the biological plausibility of inflammatory modulation in RRI susceptibility while reinforcing that no single biomarker captures sufficient variance to enable clinical prediction.

From a practical standpoint, these results are hypothesis‐generating and highlight the need for longitudinal studies with serial inflammatory measurements, integration of multiple biomarkers with training and biomechanical variables in comprehensive prediction models, and investigation of whether interventions targeting chronic inflammation might reduce injury incidence. While baseline TNF‐α measurement is unlikely to transform clinical practice in isolation, understanding inflammatory contributions to injury susceptibility may inform more effective injury prevention strategies.

## 7 Perspectives

The identification of elevated TNF‐α levels as a potential biomarker presents a valuable opportunity for screening runners who may be at increased susceptibility to overuse injuries, thereby supporting early intervention strategies in sports medicine. Furthermore, the recognition that history of musculoskeletal trauma and higher weekly running volumes serve as strong predictors of injury risk underscores the importance of incorporating these factors into individualized training load management and post‐injury return‐to‐sport protocols. However, the limited predictive value of inflammatory markers alone highlights a critical need for more comprehensive, multidisciplinary injury prevention models that integrate physiological, biochemical, and biomechanical data to better understand the complex etiology of running‐related injuries and develop more effective prevention strategies.

## Author Contributions

Lukas Cipryan, codesigned the study; analyzed and interpreted the data; drafted, revised, and submitted the manuscript. Martina Litschmannova analyzed and interpreted data; revised the manuscript. Jiri Skypala, Tomas Dostal, Dominik Sindler collected data and revised the manuscript, Daniel Jandacka (principal investigator of the 4HAIE and LERCO projects) designed the study, interpreted data, and revised the manuscript, Peter Hofmann interpreted data and revised the manuscript. All authors approved the final version of the manuscript.

## Funding

This work has been produced with the financial support of the European Union under the LERCO project (CZ.10.03.01/00/22_003/0000003) via the Operational Programme Just Transition and research related to wellbeing from the project Research of Excellence on Digital Technologies and Wellbeing CZ.02.01.01/00/22_008/0004583 which is co‐financed by the European Union. Statistical analysis was partially supported by Grant of SGS No. SP2025/012, VŠB‐Technical University of Ostrava, Czech Republic. The baseline data refers to the project funded by the Ministry of Education, Youth and Sports, Czech Republic, the project 4HAIE “Healthy Aging in the Industrial Environment‐Program 4” (CZ.02.1.01/0.0/0.0/16_019/0000798) within its sustainability period.

## Conflicts of Interest

The authors declare no conflicts of interest.

## Supporting information


**Appendix S1:** Supporting Information.

## Data Availability

The data that support the findings of this study are available from the corresponding author upon reasonable request.

## References

[1] Z. Pedisic , N. Shrestha , S. Kovalchik , et al., “Is Running Associated With a Lower Risk of All‐Cause, Cardiovascular and Cancer Mortality, and Is the More the Better? A Systematic Review and Meta‐Analysis,” British Journal of Sports Medicine 54 (2020): 898–905. 31685526 10.1136/bjsports-2018-100493

[2] P. Oja , A. R. Memon , S. Titze , et al., “Health Benefits of Different Sports: A Systematic Review and Meta‐Analysis of Longitudinal and Intervention Studies Including 2.6 Million Adult Participants,” Sports Medicine 10 (2024): 46. 10.1186/s40798-024-00692-x PMC11043276 38658416

[3] M. Rahmati , H. Lee , H. Lee , et al., “Associations Between Exercise Training, Physical Activity, Sedentary Behaviour and Mortality: An Umbrella Review of Meta‐Analyses,” Journal of Cachexia, Sarcopenia and Muscle 16 (2025): e13772. 40042073 10.1002/jcsm.13772 PMC11880915

[4] I. Buist , S. W. Bredeweg , B. Bessem , W. van Mechelen , K. A. P. M. Lemmink , and R. L. Diercks , “Incidence and Risk Factors of Running‐Related Injuries During Preparation for a 4‐Mile Recreational Running Event,” British Journal of Sports Medicine 44 (2010): 598–604. 18487252 10.1136/bjsm.2007.044677

[5] J. Schuster Brandt Frandsen , A. Hulme , E. T. Parner , et al., “How Much Running Is Too Much? Identifying High‐Risk Running Sessions in a 5200‐Person Cohort Study,” British Journal of Sports Medicine 59 (2025): 1203–1210. 40623829 10.1136/bjsports-2024-109380 PMC12421110

[6] N. Kakouris , N. Yener , and D. T. P. Fong , “A Systematic Review of Running‐Related Musculoskeletal Injuries in Runners,” Journal of Sport and Health Science 10 (2021): 513–522. 33862272 10.1016/j.jshs.2021.04.001 PMC8500811

[7] S. Docherty , R. Harley , J. J. McAuley , et al., “The Effect of Exercise on Cytokines: Implications for Musculoskeletal Health: A Narrative Review,” BMC Sports Science, Medicine and Rehabilitation 14 (2022): 5. 10.1186/s13102-022-00397-2 PMC8740100 34991697

[8] M. Cifuentes , H. E. Verdejo , P. F. Castro , et al., “Low‐Grade Chronic Inflammation: A Shared Mechanism for Chronic Diseases,” Physiology (Bethesda) 40 (2025): 592. 10.1152/physiol.00021.2024 39078396

[9] F. A. Orlando and A. G. Mainous , “Editorial: Inflammation and Chronic Disease,” Frontiers in Medicine (Lausanne) 11 (2024): 1434533. 10.3389/fmed.2024.1434533 PMC11249529 39015780

[10] R. Medzhitov , “Inflammation 2010: New Adventures of an Old Flame,” Cell 140 (2010): 771–776. 20303867 10.1016/j.cell.2010.03.006

[11] E. K. Merritt , M. J. Stec , A. Thalacker‐Mercer , et al., “Heightened Muscle Inflammation Susceptibility May Impair Regenerative Capacity in Aging Humans,” Journal of Applied Physiology (1985) 115 (2013): 937–948. 10.1152/japplphysiol.00019.2013 PMC3764621 23681911

[12] I. Stratos , A. K. Behrendt , C. Anselm , A. Gonzalez , T. Mittlmeier , and B. Vollmar , “Inhibition of TNF‐α Restores Muscle Force, Inhibits Inflammation, and Reduces Apoptosis of Traumatized Skeletal Muscles,” Cells 11 (2022): 2397. 35954240 10.3390/cells11152397 PMC9367740

[13] P. Berraondo , M. F. Sanmamed , M. C. Ochoa , et al., “Cytokines in Clinical Cancer Immunotherapy,” British Journal of Cancer 120 (2019): 6–15. 30413827 10.1038/s41416-018-0328-y PMC6325155

[14] K. Papanikolaou , D. Draganidis , I. G. Fatouros , and A. Z. Jamurtas , “Inflammatory Response to Different Types of Exercise,” in Fundamentals of Recovery, Regeneration, and Adaptation to Exercise Stress: An Integrated Approach (Springer Nature Switzerland, 2025), 213–234.

[15] C. K. Correia , J. M. Machado , F. H. Dominski , M. P. de Castro , H. Brito Fontana , and C. Ruschel , “Risk Factors for Running‐Related Injuries: An Umbrella Systematic Review,” Journal of Sport and Health Science 13 (2024): 793–804. 38697289 10.1016/j.jshs.2024.04.011 PMC11336318

[16] J. Plesek , J. Hamill , M. Burda , et al., “Running Distance and Biomechanical Risk Factors for Plantar Fasciitis: A 1‐Yr Prospective 4HAIE Cohort Study,” Medicine and Science in Sports and Exercise 57 (2025): 756–766. 39629715 10.1249/MSS.0000000000003617 PMC11878588

[17] L. Cipryan , P. Kutac , T. Dostal , et al., “Regular Running in an Air‐Polluted Environment: Physiological and Anthropometric Protocol for a Prospective Cohort Study (Healthy Aging in Industrial Environment Study – Program 4),” BMJ Open 10 (2020): e040529. 10.1136/bmjopen-2020-040529 PMC7733192 33303450

[18] D. Jandacka , J. Uchytil , D. Zahradnik , et al., “Running and Physical Activity in an Air‐Polluted Environment: The Biomechanical and Musculoskeletal Protocol for a Prospective Cohort Study 4HAIE (Healthy Aging in Industrial Environment—Program 4),” International Journal of Environmental Research and Public Health 17 (2020): 9142. 33297585 10.3390/ijerph17239142 PMC7730319

[19] S. Elavsky , V. Jandačková , L. Knapová , et al., “Physical Activity in an Air‐Polluted Environment: Behavioral, Psychological and Neuroimaging Protocol for a Prospective Cohort Study (Healthy Aging in Industrial Environment Study – Program 4),” BMC Public Health 21 (2021): 126. 33435943 10.1186/s12889-021-10166-4 PMC7801866

[20] M. L. Bertelsen , A. Hulme , J. Petersen , et al., “A Framework for the Etiology of Running‐Related Injuries,” Scandinavian Journal of Medicine & Science in Sports 27 (2017): 1170–1180. 28329441 10.1111/sms.12883

[21] D. Riebe , ed., Guidelines for Exercise Testing and Prescription (ACSM) (Wolters Kluwer Health | Lippincott Williams & Wilkins, 2018).

[22] V. A. B. Costa , A. W. Midgley , S. Carroll , et al., “Is a Verification Phase Useful for Confirming Maximal Oxygen Uptake in Apparently Healthy Adults? A Systematic Review and Meta‐Analysis,” PLoS One 16 (2021): e0247057. 33596256 10.1371/journal.pone.0247057 PMC7888616

[23] P. Birnbaumer , T. Dostal , L. Cipryan , and P. Hofmann , “Pattern of the Heart Rate Performance Curve in Maximal Graded Treadmill Running From 1100 Healthy 18–65 Years Old Men and Women: The 4HAIE Study,” Frontiers in Physiology 14 (2023): 1–10. 10.3389/fphys.2023.1178913 PMC10264846 37324398

[24] K. Wiegand , J. A. Mercer , J. W. Navalta , J. Pharr , R. Tandy , and J. Freedman Silvernail , “Running Status and History: A Self‐Report Study,” Physical Therapy in Sport 39 (2019): 8–15. 31202143 10.1016/j.ptsp.2019.06.003

[25] T. P. Yamato , B. T. Saragiotto , and A. D. Lopes , “A Consensus Definition of Running‐Related Injury in Recreational Runners: A Modified Delphi Approach,” Journal of Orthopaedic & Sports Physical Therapy 45 (2015): 375–380. 25808527 10.2519/jospt.2015.5741

[26] R. Ø. Nielsen , M. L. Bertelsen , D. Ramskov , et al., “The Garmin‐RUNSAFE Running Health Study on the Aetiology of Running‐Related Injuries: Rationale and Design of an 18‐Month Prospective Cohort Study Including Runners Worldwide,” BMJ Open 9 (2019): e032627. 10.1136/bmjopen-2019-032627 PMC6731941 31494626

[27] B. T. Saragiotto , T. P. Yamato , L. C. Hespanhol Junior , M. J. Rainbow , I. S. Davis , and A. D. Lopes , “What Are the Main Risk Factors for Running‐Related Injuries?,” Sports Medicine 44 (2014): 1153–1163. 24809248 10.1007/s40279-014-0194-6

[28] A. A. Gharamti , O. Samara , A. Monzon , et al., “Proinflammatory Cytokines Levels in Sepsis and Healthy Volunteers, and Tumor Necrosis Factor‐Alpha Associated Sepsis Mortality: A Systematic Review and Meta‐Analysis,” Cytokine 158 (2022): 156006. 36044827 10.1016/j.cyto.2022.156006

[29] R. L. Starkie , J. Rolland , D. J. Angus , M. J. Anderson , and M. A. Febbraio , “Circulating Monocytes Are Not the Source of Elevations in Plasma IL‐6 and TNF‐α Levels After Prolonged Running,” American Journal of Physiology‐Cell Physiology 280 (2001): C769–C774. 11245592 10.1152/ajpcell.2001.280.4.C769

[30] P. K. Langston and D. Mathis , “Immunological Regulation of Skeletal Muscle Adaptation to Exercise,” Cell Metabolism 36 (2024): 1175–1183. 38670108 10.1016/j.cmet.2024.04.001 PMC11153001

[31] H. Tu and Y. L. Li , “Inflammation Balance in Skeletal Muscle Damage and Repair,” Frontiers in Immunology 14 (2023): 133355. 10.3389/fimmu.2023.1133355 PMC9909416 36776867

[32] D. Benezra , G. Maftzir , and V. Barak , “Blood Serum Interleukin‐1 Receptor Antagonist in Pars Planitis and Ocular Behçet Disease,” American Journal of Ophthalmology 123 (1997): 593–598. 9152064 10.1016/s0002-9394(14)71071-2

[33] X. Liao , Y. Xiao , U. Elbelt , et al., “Association of Interleukin‐1 Beta and Interleukin‐1 Receptor Antagonist Gene Polymorphisms and Plasma Levels With Diabetic Nephropathy,” BioMed Research International 2022 (2022): 9661823. 35663044 10.1155/2022/9661823 PMC9159863

[34] M. Hurme and S. Santtila , “IL‐1 Receptor Antagonist (IL‐1Ra) Plasma Levels Are Co‐Ordinately Regulated by Both IL‐1Ra and IL‐1beta Genes,” European Journal of Immunology 28 (1998): 2598–2602. 9710237 10.1002/(SICI)1521-4141(199808)28:08<2598::AID-IMMU2598>3.0.CO;2-K

[35] S. M. O'Brien , P. Fitzgerald , P. Scully , A. Landers , L. V. Scott , and T. G. Dinan , “Impact of Gender and Menstrual Cycle Phase on Plasma Cytokine Concentrations,” Neuroimmunomodulation 14 (2007): 84–90. 17713355 10.1159/000107423

[36] G. L. Reyneke , K. Lambert , and E. J. Beck , “Dietary Patterns Associated With Anti‐Inflammatory Effects: An Umbrella Review of Systematic Reviews and Meta‐Analyses,” Nutrition Reviews (2025): nuaf104, Online ahead of print, 10.1093/nutrit/nuaf104. 40657695 PMC13161761

[37] S. Quarta , M. Massaro , M. A. Carluccio , et al., “An Exploratory Critical Review on TNF‐α as a Potential Inflammatory Biomarker Responsive to Dietary Intervention With Bioactive Foods and Derived Products,” Food 11 (2022): 2524. 10.3390/foods11162524 PMC9407274 36010524

