# Supplementary material for: Baseline Inflammatory Markers as Predictors of Running‐Related Injuries: A One‐Year Prospective 4HAIE Cohort Study
Source: Scand J Med Sci Sports. 2026 Feb 12;36(2):e70225. doi: 10.1111/sms.70225 (PMC12895214; doi:10.1111/sms.70225)
Supplement: Supplementary file 1 — Appendix S1: Supporting Information. [file SMS-36-e70225-s001.docx]

**Baseline inflammatory markers as predictors of running-related injuries: A one-year prospective 4HAIE cohort study**

Lukas Cipryan, Jiri Skypala, Martina Litschmannova, Daniel Jandacka, Tomas Dostal, Dominik Sindler, David Zahradnik, Peter Hofmann

Supplemental Material

**Table S1. Basic characteristics of the cohort** (N = 1,315; male/female: 706 (53.7%)/609 (46.3%)).

|  | **(min; max)** | **M (Q1; Q3)** |
| --- | --- | --- |
| Age (years) | (18.0; 65.0) | 39.0 (27.0; 47.0) |
| Height (cm) | (148.5; 201.6) | 174.4 (167.9; 181.2) |
| Body mass (kg) | (40.5; 132.3) | 74.2 (64.5; 84.9) |
| BMI (kg/m^2^) | (16.0; 49.4) | 24.1 (21.9; 26.) |
| Total body fat (%) | (3.5; 53.1) | 20.9 (15.5; 28.2) |
| VO_2peak_ (ml/kg/min) | (14.6; 70.9) | 41.8 (34.6; 49.2) |
| VO_2peak_ (l/min) | (1.14; 5.67) | 3.00 (2.39; 3.81) |
| RER (-) | (0.62; 2.23) | 1.12 (1.07; 1.16) |
| Running distance (km/week) | (0.1; 95.2) | 5.3 (0.7; 13.8) |
| Systolic BP (mmHg) | (84.0; 180.0) | 127.0 (117.0; 137.0) |
| Diastolic BP (mmHg) | (45.3; 111.7) | 79.0 (72.3; 86.3) |
| IL-1β (pg/ml) | (0.00; 73.00) | 0.39 (0.19; 0.51) |
| IL-1RA (pg/ml) | (0.0; 6,235.1) | 466.7 (344.4; 624.1) |
| IL-6 (pg/ml) | (0.00; 145.77) | 0.17 (0.07; 0.54) |
| IL-10 (pg/ml) | (0.00; 64.47) | 0.12 (0.05; 0.40) |
| TNF-α (pg/ml) | (0.00; 10.89) | 0.91 (0.38; 1.58) |
| CRP (mg/l) | (0.00; 55.10) | 0.50 (0.50; 1.70) |
| Fibrinogen (g/l) | (0.00; 5.00) | 2.63 (2.31; 2.97) |
| Adiponectin (ng/ml) | (0; 10,6189) | 5,928 (3,700; 8,913) |
| Leptin (pg/ml) | (0; 11,2897) | 6,210 (2,456; 12,509) |
| Adiponectin/Leptin (-) | (0.00; 8,570.14) | 0.96 (0.40; 2.52) |
| BDNF (pg/ml) | (0; 14,2792) | 24,171(18,973; 29,710) |

Legend: M – Median, Q1/Q3 – quartile 1 and 3, BMI – body mass index, VO_2peak_ – peak oxygen consumption, BP – blood pressure, IL-1β – interleukin 1β, IL-1RA – interleukin 1 receptor antagonist, IL-6 – interleukin 6, IL-10 – interleukin 10, TNF-α – tumor necrosis factor α, CRP – C-reactive protein, BDNF – brain derived neurotrophic factor.

**Table S2.** **Initial multivariate logistic regression model (Model 0).**

| **Predictors** | **Coef** | **SE(Coef)** | **p-value** | **OR (95% CI)** |
| --- | --- | --- | --- | --- |
| Intercept | -2.202 | 0.824 | **0.008** | --- |
| Age (years) | 0.017 | 0.008 | **0.022** | 1.02 (1.00; 1.03) |
| Sex (male/female) | -0.027 | 0.174 | 0.876 | 0.97 (0.69; 1.37) |
| Running distance (km/week) | 0.019 | 0.006 | **0.002** | 1.02 (1.00; 1.03) |
| Retrospective MST (Yes/No) | 0.441 | 0.141 | **0.002** | 1.56 (1.18; 2.06) |
| VO_2peak_ (ml/kg/min) | 0.013 | 0.012 | 0.284 | 1.01 (0.98; 1.04) |
| IL-1β (pg/ml) | -0.055 | 0.055 | 0.312 | 0.94 (0.81; 1.02) |
| IL-1RA (pg/ml) | -0.001 | <0.001 | **0.033** | 1.00 (1.00; 1.00) |
| IL-6 (pg/ml) | 0.039 | 0.041 | 0.339 | 1.04 (1.00; 1.18) |
| IL-10 (pg/ml) | -0.081 | 0.100 | 0.417 | 0.92 (0.70; 1.02) |
| TNF-α (pg/ml) | 0.257 | 0.079 | **0.001** | 1.29 (1.11; 1.51) |
| CRP (mg/l) | 0.002 | 0.031 | 0.950 | 1.00 (0.93; 1.06) |
| Fibrinogen (g/l) | -0.155 | 0.167 | 0.352 | 0.86 (0.61; 1.19) |
| Adiponectin (ng/ml) | <0.001 | <0.001 | 0.295 | 1.00 (1.00; 1.00) |
| Leptin (pg/ml) | <0.001 | <0.001 | 0.572 | 1.00 (1.00; 1.00) |
| Adiponectin/Leptin (-) | 0.001 | 0.001 | 0.241 | 1.00 (1.00; 1.00) |
| BDNF (pg/ml) | <0.001 | <0.001 | 0.838 | 1.00 (1.00; 1.00) |
| n = 1159, R^2^ = 0.075, AIC = 1303.2 | | | | |

Legend: OR – odds ratio, CI – confidence interval, IL-1β – interleukin 1β, IL-1RA – interleukin 1 receptor antagonist, IL-6 – interleukin 6, IL-10 – interleukin 10, TNF-α – tumor necrosis factor α, CRP – C-reactive protein, BDNF – brain derived neurotrophic factor.

**Table S3. Group comparison: retrospectively reported MST and RRI (N = 1,315).**

|  |  | **Retrospective MST** | | **Total**  **(n = 1 315)** | **OR (95% CI)** | **Fisher's test (p)** |
| --- | --- | --- | --- | --- | --- | --- |
|  |  | **Yes**  **(n = 516)** | **No**  **(n = 799)** |  |  |  |
| **RRI (Model 1)** | **Yes** | 166 (32.2) | 176 (22.0) | 342 (26.0) | 1.68  (1.29. 2.18) | < 0.001 |
| **ALL_RRI_MDD_One_Year** | **No** | 350 (67.8) | 623 (78.0) | 973 (74.0) |  |  |
| **Self-reported RRI** | **Yes** | 116 (22.5) | 118 (14.8) | 234 (17.8) | 1.67  (1.24. 2.25) | 0.001 |
| **ALL_RRI_One_Year** | **No** | 400 (77.5) | 681 (85.2) | 1081 (82.2) |  |  |
| **Medically confirmed RRI** | **Yes** | 93 (18.0) | 94 (11.8) | 187 (14.2) | 1.65  (1.19. 2.28) | 0.001 |
| **ALL_MDD_One_Year** | **No** | 423 (82.0) | 705 (88.2) | 1128 (85.8) |  |  |
| **RRI over 30 days after baseline** | **Yes** | 45 (8.7) | 54 (6.8) | 99 (7.5) | 1.32  (0.85. 2.04) | 0.200 |
| **ALL_RRI_MDD_30days** | **No** | 471 (91.3) | 745 (93.2) | 1216 (92.5) |  |  |

Legend: RRI – running related injury, MST – musculoskeletal trauma, OR – odds ratio, CI – confidence interval

**Table S4. Group comparison: retrospectively reported MST and RRI (N = 1,159).**

|  |  | **Retrospective MST** | | **Total**  **(n = 1,159)** | **OR (95% CI)** | **Fisher's test (p)** |
| --- | --- | --- | --- | --- | --- | --- |
|  |  | **Yes**  **(n = 314)** | **No**  **(n = 845)** |  |  |  |
| **RRI (Model 1)** | **Yes** | 153 (33.8) | 299 (22.8) | 452 (39.0) | 1.73  (1.32. 2.28) | < 0.001 |
| **ALL_RRI_MDD_One_Year** | **No** | 161 (66.2) | 546 (77.2) | 707 (61.0) |  |  |
| **Self-reported RRI** | **Yes** | 106 (23.5) | 109 (15.4) | 215 (18.6) | 1.68  (1.23. 2.29) | 0.001 |
| **ALL_RRI_One_Year** | **No** | 346 (76.5) | 598 (84.6) | 944 (81.4) |  |  |
| **Medically confirmed RRI** | **Yes** | 86 (19.0) | 85 (12.0) | 171 (14.8) | 1.72  (1.22. 2.42) | 0.001 |
| **ALL_MDD_One_Year** | **No** | 366 (81.0) | 622 (88.0) | 988 (85.2) |  |  |
| **RRI over 30 days after baseline** | **Yes** | 41 (9.1) | 48 (6.8) | 89 (7.7) | 1.37  (0.86. 2.16) | 0.174 |
| **ALL_RRI_MDD_30days** | **No** | 411 (90.9) | 659 (93.2) | 1,070 (92.3) |  |  |

Legend: RRI – running related injury, MST – musculoskeletal trauma, OR – odds ratio, CI – confidence interval
